# Farming fit? Dispelling the Australian agrarian myth

**DOI:** 10.1186/1756-0500-4-89

**Published:** 2011-03-30

**Authors:** Susan Brumby, Ananda Chandrasekara, Scott McCoombe, Peter Kremer, Paul Lewandowski

**Affiliations:** 1 National Centre for Farmer Health, Western District Health Service, Hamilton Vic 3300, Australia; 2 School of Medicine, Deakin University, Geelong Vic 3217, Australia; 3 School of Psychology, Deakin University, Geelong Vic 3217, Australia

## Abstract

**Background:**

Rural Australians face a higher mental health and lifestyle disease burden (obesity, diabetes and cardiovascular disease) than their urban counterparts. Our ongoing research reveals that the Australian farming community has even poorer physical and mental health outcomes than rural averages. In particular, farm men and women have high rates of overweightness, obesity, abdominal adiposity, high blood pressure and psychological distress when compared against Australian averages. Within our farming cohort we observed a significant association between psychological distress and obesity, abdominal adiposity and body fat percentage in the farming population.

**Presentation of hypothesis:**

This paper presents a hypothesis based on preliminary data obtained from an ongoing study that could potentially explain the complex correlation between obesity, psychological distress and physical activity among a farming population. We posit that spasmodic physical activity, changing farm practices and climate variability induce prolonged stress in farmers. This increases systemic cortisol that, in turn, promotes abdominal adiposity and weight gain.

**Testing the hypothesis:**

The hypothesis will be tested by anthropometric, biochemical and psychological analysis matched against systemic cortisol levels and the physical activity of the subjects.

**Implications of the hypothesis tested:**

Previous studies indicate that farming populations have elevated rates of psychological distress and high rates of suicide. Australian farmers have recently experienced challenging climatic conditions including prolonged drought, floods and cyclones. Through our interactions and through the media it is not uncommon for farmers to describe the effect of this long-term stress with feelings of 'defeat'. By gaining a greater understanding of the role cortisol and physical activity have on mental and physical health we may positively impact the current rates of psychological distress in farmers.

**Trial registration:**

ACTRN12610000827033

## Background

The agrarian myth of an agricultural utopia supports the notion that living on a farm is coupled to the stereotypical view of stress-free, happy and healthy lifestyle. Unfortunately, this is not the case as rural Australians face an environment of high occupational hazards, poor access to services, higher mental health burden, vulnerability to adverse climatic conditions, socio-economic constraints, food insecurity, alcohol misuse and an increasing burden of chronic disease[1].

In particular, Australian farmers are at higher risk of physical and mental illness when compared to rural and metropolitan populations. These differences are highlighted by the increased incidence of suicide in farming communities[2]. Suicide rates are linked to the larger underlying issues of social isolation, stigma, poor access to health services and the prevailing attitudes and beliefs of farming communities.

It is widely reported that there are multifarious interrelationships between lifestyle, anthropometric, psychological and activity variables [3]. Depressed or chronically stressed people generally have lower levels of physical activity than people without mental illness [4]. Additionally, lower physical activity levels can exacerbate the effects of depression, anxiety and stress, thereby resulting in a downward spiral towards poorer health. Conversely, increasing physical activity has the benefits of not only increasing physical fitness but also alleviating depression, stress and anxiety[5].

### Preliminary study results

The Sustainable Farm Families™ (SFF) program focuses on the health, well-being and safety of farm men and women [6]. This on going service delivery and research program has enabled key insights to be made into both the magnitude, and causes, of poor health within the Australian agricultural community.

**Figure 1:**
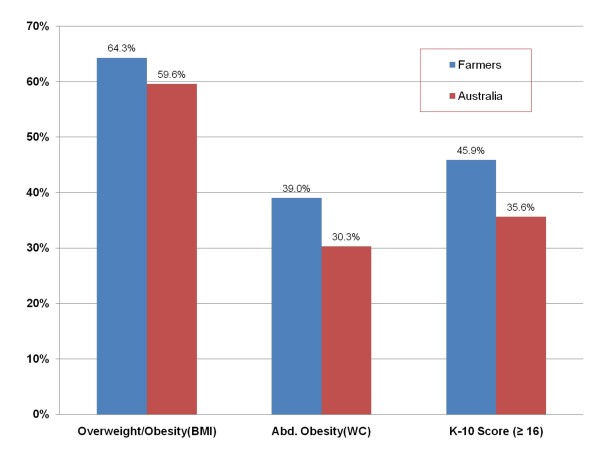
**Graphical representation of national and farming cohort prevalence's (%) of body mass index (BMI) ≥ 25 (kg/m^2^), at-risk waist circumference (WC) measurement (>88 cm for females, >102 cm for males) and high Kessler-10 score (≥16) **[7,8]. The age standardised farmer data was collected during an ongoing cross sectional study of farming populations.

A preliminary analysis of SFF data was conducted on a cohort of 1813 adult farmers, revealing that physical and mental health indicators are substandard within farming communities. This preliminary analysis also revealed that 64.3% of farm men and women were either obese or overweight which is significantly higher than current age standardised Australian national averages (p < 0.05) (Figure 1). The prevalence of abdominal obesity (waist circumference >88 cm for females and >102 cm for males) in this study group was 8.7% higher than the Australian national population[7].

In light of the established research linking mental well-being to physical activity [5], we decided to investigate whether farmer obesity was linked to psychological distress within our cohort. Our findings revealed that farmers have higher rates of psychological distress (45.9% with Kessler K.10 score ≥ 16 [7]) than rural Victorians (31.3%), state (32.9%) or national (35.6%) averages[8]. Furthermore, Table 1 reveals a significant association between psychological distress and abdominal adiposity (waist circumference), obesity (Body Mass Index) and high body fat percentages (measured using single beam bioelectrical impedance analysis[9]) in mature aged (≥50 years of age) participants.

**Table 1:** The relationship of apparent psychological distress and anthropometric characteristics in mature aged farm men and women (≥50 years old)

		Not distressed n (%)	**Psychologically distressed**^a ^**n (%)**	P
**Body Mass Index**	Obese/overweight	273(68.4)	222(77.4)	0.010*
	
	Not obese	126(31.6)	65(22.6)	

**Abdominal obesity**	Obese	239(61.1)	202(72.4)	0.02*

**(Waist circumference**)	Not obese	152(38.9)	77(27.6)	

**Body fat%**	High body fat%	230(63.2)	194(71.9)	0.022*
	
	Not high body fat%	134(36.8)	76(28.1)	

^a ^Psychological distress was assumed for K-10 score ≥16 or previously diagnosed mental illness. Probability (p) calculated using two-tail Pearson chi-square test. * Significance was assumed if p ≤ 0.05

In our preliminary analysis of farmer mental and physical health, we utilised the Pearson chi-square test to identify the interrelationship of psychological distress status and the prevalence of obesity (body mass index), abdominal obesity (waist circumference) and body fat percentage (measured using single beam bioelectrical impedance analysis [9]).

Farmers participating in this preliminary cohort have described the feeling of being unable to make decisions, their lives being 'on pause' following 7-8 years of drought and cite further stress caused by reduced health, government and financial services [9]. Additionally, both male and female farmers have reduced their physical activity levels due to decreased farming production, less livestock, fewer recreational activities and increased mechanisation[10].

It is well documented that stress over an extended period can result in an individual becoming chronically distressed and experiencing a feeling of hopelessness[11]. This is known as the 'defeat' response and is characterised by an increase in circulating cortisol[11]. Changes in circulating cortisol levels have been associated with psychological stress, depression, illness, trauma and physical exertion. Cortisol also regulates abdominal fat deposition, visceral obesity, and suppression of the immune system and if chronically raised can lead to diseases such as obesity and diabetes[11].

### Presentation of the hypothesis

Based on our preliminary cross sectional analysis, the established links between physical activity and mental well-being and the 'defeat' response, we hypothesise that the prolonged levels of stress experienced by farm men and women increases their exposure to the hormone cortisol.

Chronic exposure to cortisol in turn adversely impacts fat storage, resulting in abdominal adiposity and weight gain and, in turn, further cortisol release. Conversely, improving physical activity positively impacts fat storage and increases endorphin concentration [12] which improves mood state and reduces circulating cortisol levels. These physical activity induced changes to the hypothalamic pituitary adrenal (HPA) axis have the dual benefit of improving the psychological state and reducing adiposity.

Drawing on these factors we propose the Farming Fit hypothesis which explores the interrelationships of these factors among the farm men and women.

### Farming Fit Hypothesis

The spasmodic level of physical activity and the prolonged stress that farm men and women experience has the potential to increase their systemic cortisol, adversely affecting fat storage and promoting weight gain. Without intervention, weight gain, mental health and exercise levels become increasingly compromised in a spiralling manner. Abdominal obesity has been linked with up regulation of the HPA axis[13,14]. HPA up regulation is a side effect of chronic stress [15] and leads to raised diurnal cortisol secretion. Abnormal regulation of the HPA axis and perceived stress-dependent cortisol values are strongly related to abdominal obesity along with metabolic abnormalities[16,17].

**Figure 2:**
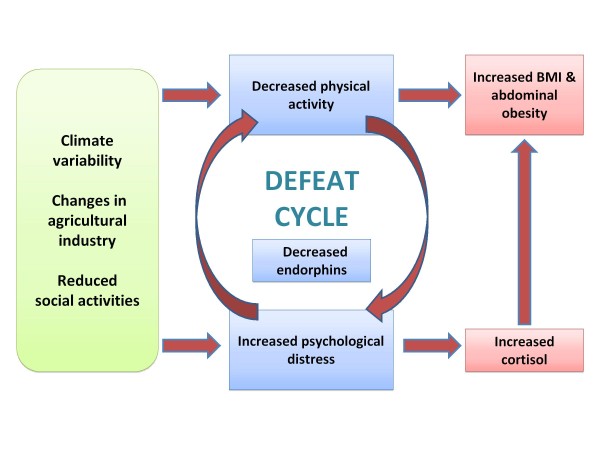
**Farming Fit hypothesis**. Preliminary data obtained from the Sustainable Farm Families program indicates that farmers are no longer as active as they used to be. The reasons for this are multi-factorial with the biggest impacts coming from increasing agricultural mechanisation, (decreased farm physical work) poor diet, decreasing local recreation activities, lifestyle health risks (including obesity, pre-diabetes and chronic pain), decreasing social opportunities and climate variability (drought, flood and extreme weather events). The decrease in physical activity has led to increased farmer overweightness and obesity and has negatively impacted mental health. Increased psychological distress and obesity both increase circulating cortisol levels and decrease circulating endorphins. In turn, this biochemical milieu further raises psychological stress and promotes fat deposition. Psychological distress decreases an individual's willingness to engage in physical activity, creating a cycle of defeat with increasing weight gain and poorer mental health. We hypothesise a farmer who is chronically stressed can intervene in this cycle by increasing physical activity which reduces body mass, decreases cortisol levels, increases endorphin release and improves psychological health.

We illustrate this hypothesis in the Farming Fit hypothesis (Figure 2). The physical and mental fitness of farm men and women are heavily impacted by climatic variability, changing agricultural practices, social isolation and increasing sedentary work and non-work practices. Recurrent conversations with farm men and women anecdotally reinforce that their physical activity is seasonal, spasmodic, unplanned or non-existent[10].

Furthermore, long-term drought, flood and extreme weather events have reduced livestock numbers, decimated cropping and production systems and subsequently lowered the physical activity demands these tasks require. Additionally rural communities have suffered population losses resulting in the closure of, or amalgamations of local sporting teams such as tennis, football or netball as well as the demise of domestic gardens all decreasing physical activity opportunities.

Improving opportunities for regular physical activity on the farm and within farming communities with a sustained and well-planned program may improve circulating cortisol levels, reduce the incidence of obesity in farm men and women and ease psychological distress.

### Testing the hypothesis

In order to test the Farming Fit hypothesis we will conduct a quasi-experimental longitudinal control intervention study, which will evaluate the effect of increasing physical activity in selected overweight/obese farm men and women. The intervention will include a personalised exercise program designed by an exercise physiologist to be undertaken with regular coaching input and support over the 6-month period. This intervention will not be made accessible to a corresponding control group until after the 6-month study.

The effect of physical activity on psychological health and biochemical levels of serum cholesterol, triglycerides, low density lipoproteins (LDL), high density lipoproteins (HDL), fasting blood glucose and cortisol will be measured in overweight and obese farm men and women.

### Measures

#### 1. Assessment

A complete health history will be obtained from participants registered in the Farming Fit study. Assessments will include anthropometric measures of height, weight, hip and waist circumference, body fat analysis and biochemical analysis of fasted total serum cholesterol, triglycerides, low density lipids (LDL), high density lipids (HDL), blood glucose and cortisol. Salivary cortisol at different time points across a 24-hour period will also be collected.

#### 2. Behavioral assessment

Alcohol habits, activity levels, dietary habits and smoking will be recorded.

#### 3. Psychosocial assessment

A validated questionnaire, the depression, anxiety, stress score (DASS) will be used. The DASS 21 is made up of three seven item subscales covering depression, anxiety and stress as specified in Lovibonds and Lovibond [18].

### Ethical approval and confidentiality

Ethics approval has been granted to the project by Deakin University Human Research Ethics Committee (HREC 2009/215 dated 03/02/2010) and the South West Multidisciplinary Ethics Committee. All participants in the study will be provided with a plain language statement to enable informed written consent prior to participation in this study. The collected data will be appropriately stored at the Western District Health Service in Hamilton, Victoria, Australia.

### Implication of the hypothesis

Rural populations face poor outcomes in mental health and the associated co-morbidities of obesity, diabetes and cardiovascular disease. Farming populations are a subset of this group that face additional challenges due to the tyranny of distance, the distance decay effect and predicted on going climatic and environmental challenges. Farmers have a high mental health burden and higher rates of obesity and associated lifestyle disease.

It is plausible that the anthropometric and psychological conditions we observed in our preliminary farming cohort are linked to this 'defeat' response [11]. However, convincing farm people that they should consider physical activity to improve either their health or their mental well-being is met with scepticism and disbelief [10].

The outcome results of this study will be used in further research into the physical and mental health of farmers as well as provide important insights in to the development of novel physical activity interventions, improved service delivery programs and future prevention opportunities.

### Limitations of hypothesis testing

The presence of confounding factors such as dietary variation, physical activity dissimilarity and individual social interaction may affect the final outcome of this study but are difficult to control. The effects of climate variability in the short term may also affect the final outcomes of the Farming Fit hypothesis but are outside the scope of this project. Further extended research on these confounding factors is warranted.

### Concluding remarks

Australian farmers experience high rates of physical and psychological disease. As global demand for food rises, Australia as a large food exporter and food manufacturer will require a healthy farming workforce to enable it to make the most of this opportunity. With depleting agricultural communities, an ageing farmer workforce and predicted climatic adversity, it is important to understand the links between physical and mental health and agricultural productivity. Understanding how physical health is important for the well-being of farmers and their enterprise will help to break the 'defeat' cycle and assist the agricultural industry to be Farming Fit.

## List of abbreviations

SFF: Sustainable Farm Families™; K-10, Kessler 10; HPA: Hypothalamus Pituitary Adrenal; LDL: Low Density Lipoproteins; HDL: High Density Lipoproteins; DASS-21: Depression, Anxiety, Stress Score-21; WC: Waist circumference; BMI: Body Mass Index.

## Competing interests

The authors declare that they have no competing interests.

## Authors' contributions

SB, AC and SM originated the Farming Fit concept and drafted the initial manuscript. All authors contributed to the methods and design of the study. All authors have read and approved the final manuscript.

## References

[B1] Wainer J Chesters J Rural mental health: Neither romanticism nor despair Australian Journal of Rural Health 2000 8 3 141 147 10.1046/j.1440-1584.2000.00304.x 11249401

[B2] Miller K Burns C Suicides on farms in South Australia, 1997-2001 Australian Journal of Rural Health 2008 16 6 327 331 10.1111/j.1440-1584.2008.01011.x 19032203

[B3] Kyrios M Moore SM Hackworth N Buzwell SA Crafti N Critchley C Hardie E The influence of depression and anxiety on outcomes after an intervention for prediabetes Med J Aust 2009 190 7 S81 5 19351299 10.5694/j.1326-5377.2009.tb02476.x

[B4] Brown WJ Ford JH Burton NW Marshall AL Dobson AJ Prospective study of physical activity and depressive symptoms in middle-aged women Am J Prev Med 2005 29 4 265 72 10.1016/j.amepre.2005.06.009 16242588

[B5] Dinas P Koutedakis Y Flouris A Effects of exercise and physical activity on depression Irish Journal of Medical Science 2010 1 7 10.1007/s11845-010-0633-9 21076975

[B6] Brumby SA Willder SJ Martin J The sustainable farm families project: changing attitudes to health Rural Remote Health 2009 9 1 1012 19292570

[B7] Dunstan D Zimmet P Welborn T Sicree R Armstrong T Atkins R Cameron A Shaw J Chadban S Diabesity and associated disorders in Australia--2000: the accelerating epidemic The Australian Diabetes, Obesity and Lifestyle Study (AusDiab) 2001 Melbourne: International Diabetes Institute

[B8] Kilkkinen A Kao-Philpot A O'Neil A Philpot B Reddy P Bunker S Dunbar J Prevalence of psychological distress, anxiety and depression in rural communities in Australia Australian Journal of Rural Health 2007 15 2 114 119 10.1111/j.1440-1584.2007.00863.x 17441820

[B9] Birchip Cropping Group Critical breaking point The effects of drought and other pressures on farming families, Final report of the twelve month study 2008

[B10] Brumby S Willder S Martin J Milking their health for all its worth? Improving the health of farming families through facilitated learning Extension Farming Systems Journal 2010 1

[B11] Dallman M Pecoraro N Akana S La Fleur S Gomez F Houshyar H Bell M Bhatnagar S Laugero K Manalo S Chronic stress and obesity: A new view of "comfort food" PNAS 2003 11696 11701 10.1073/pnas.1934666100 12975524 PMC208820

[B12] Schwarz L Kindermann W Changes in B-Endorphin levels in response to aerobic and anaerobic exercise Sports Med 1992 13 1 25 36 10.2165/00007256-199213010-00003 1553453

[B13] Rosmond R Dallman M Bjorntorp P Stress-related cortisol secretion in men: relationships with abdominal obesity and endocrine, metabolic and hemodynamic abnormalities Journal of Clinical Endocrinology & Metabolism 1998 83 6 1853 10.1210/jcem.83.6.4843 9626108

[B14] Bjorntorp P Neuroendocrine factors in obesity Journal of Endocrinology 1997 155 2 193 10.1677/joe.0.1550193 9415045

[B15] Björntorp P The regulation of adipose tissue distribution in humans International Journal of Obesity 1996 20 4 291 302 8680455

[B16] Wüst S Federenko I Hellhammer D Kirschbaum C Genetic factors, perceived chronic stress, and the free cortisol response to awakening Psychoneuroendocrinology 2000 25 7 707 720 10938450 10.1016/s0306-4530(00)00021-4

[B17] Mohamed-Ali V Pinkney J Coppack S Adipose tissue as an endocrine and paracrine organ International Journal of Obesity 1998 22 1145 1158 10.1038/sj.ijo.0800770 9877249

[B18] Lovibond P Lovibond S The structure of negative emotional states: Comparison of the Depression Anxiety Stress Scales (DASS) with the Beck Depression and Anxiety Inventories Behaviour Research and Therapy 1995 33 3 335 343 10.1016/0005-7967(94)00075-U 7726811

